# Supplementary material for: COVID-19 onset reduced the sex ratio at birth in South Africa
Source: PeerJ. 2022 Aug 29;10:e13985. doi: 10.7717/peerj.13985 (PMC9435519; doi:10.7717/peerj.13985)
Supplement: Table S1 [file peerj-10-13985-s001.docx]

**Table S1. Sex ratio at birth restricted from 2013-2016.**

| **Period** | **Male live births** | **Female live births** | **Sex ratio at birth** | **95% CI** |
| --- | --- | --- | --- | --- |
| August 2013 + 2014 | 87,302 | 85,901 | 0.504 | 0.502-0.506 |
| August 2015 | 40,335 | 39,777 | 0.503 | 0.500-0.507 |
|  | Pearsons’s Chi-squared = 0.0691  P = 0.793 | |  |  |
| January 2014 + 2015 | 90,339 | 88,510 | 0.505 | 0.503- 0.507 |
| January 2016 | 39,756 | 38,441 | 0.508 | 0.505-0.512 |
|  | Pearsons’s Chi-squared = 2.3632  P = 0.124 | |  |  |

CI – confidence interval
